# Posttraumatic stress disorder in a war-affected area of Northeast Ethiopia: a cross-sectional study

**DOI:** 10.1186/s12888-023-05116-w

**Published:** 2023-08-28

**Authors:** Zelalem Birhan, Yonas Deressa, Maregu Shegaw, Sintayehu Asnakew, Tesfa Mekonen

**Affiliations:** 1 Department of Psychiatry, Wollo University College of Medicine and Health Science, Dessie, Ethiopia; 2 Department of Psychiatry, Bahir Dar University, Bahir Dar, Ethiopia; 3 School of Computing and Information Technology, University of Wollongong, Wollongong, Australia; 4 Department of Psychiatry, Debre Tabor University College of Medicine and Health Science, Debre Tabor, Ethiopia; 5 School of Psychology, The University of Queensland, Brisbane, Australia

**Keywords:** Post-traumatic stress disorder, War, War affected area

## Abstract

**Background:**

Post-Traumatic Stress Disorder (PTSD) is a chronic condition that affects a significant proportion of war survivors following war and conflict. If PTSD is not managed, it can lead to decreased quality of life and impairments in daily functioning and lead to death. This study aimed to assess the prevalence of post-traumatic stress disorder and its associated factors among residents in a war-affected area, Dessie Town, Northeast Ethiopia.

**Methods:**

A community-based cross-sectional study was conducted among adult residents in the war-affected area, Dessie Town. A total of 615 individuals were selected by a systematic random sampling method. PTSD was assessed using the Post-Traumatic Stress Disorder Checklist, Civilian Version. Multivariable logistic regressions were used to measure the associated factors. Associations between variables were described using odds ratios, 95% confidence intervals, and a p-value less than 0.05.

**Results:**

The prevalence of PTSD was 34.5% (95% CI: 31–38). Female sex (AOR: 1.82; CI: 1.18–2.82), divorced or widowed (AOR: 2.12, CI: 1.23–3.66), having only primary schooling (AOR: 2.17; CI: 1.25–3.78), depression (AOR: 2.03; CI: 1.34–3.08), experienced ill health without medical care during the wartime (AOR: 2.97; CI: 1.43–6.16), forced separation from family (AOR: 1.90; CI: 1.16–3.12), and experienced stressful life events (AOR: 1.60; CI: 1.06–2.42) were significantly associated with PTSD.

**Conclusion:**

A significant rate of PTSD was found among residents of the war-affected area, Dessie Town. One in three people was experiencing PTSD. As a result, post-war mental health early screening and intervention is a priority, particularly for females, those who are separated or divorced, and those who have experienced stressful life events due to the war.

## Background

Post-Traumatic Stress Disorder (PTSD) is a mental disorder that can develop after experiencing or witnessing the actual or threatened loss of life or severe damage [1]. PTSD results in a response of fear manifested by recurring memories and dreams, avoidance of trauma-related stimuli, increased arousal, and negative changes in cognition that emerge after a month of occurrence [1].

PTSD affects nearly 4% of the world’s population, making it a significant contributor to the global disease burden that lasts over a year in half of all cases [2]. It is more common in war-torn communities than in areas without a recent history of open combat [3]. In 2019, about 227 million adult war survivors globally experienced PTSD, which could have debilitating consequences for their mental health and remain chronic for many years after the war if left untreated [4]. PTSD is linked to substantial repercussions, including a decreased quality of life and greater use of health and social services [5].

A recent study that included a systematic literature review and meta-analysis found that about 23.81% of adult war survivors living in post-war regions, particularly those in low-to-middle-income countries, suffer from PTSD. This equates to approximately 242 million people [6].

PTSD is thought to affect 5–6% of men and 10–12% of women in the general populations [7]. In a study conducted seven years after the conflict in three districts in northern Uganda, the prevalence of PTSD was 11.8% [8], 62.1% in Kenya on post-election violence IDP survivors by using a cross-sectional study design [9], 28% in a cross-sectional community study in South Sudan’s war-affected population [10], and 37.3% of the survivors had PTSD among Koshe landslide survivors in Addis Ababa, Ethiopia [11].

The national and regional magnitude of PTSD in Sub-Saharan Africa war and armed conflict-exposed regions is as high as 30% [12]. PTSD causes a multidimensional impact on the mental, physical, and psychosocial well-being of war survivors, leading to debilitating functional impairment and poor health-related quality of life [13, 14]. The most common mental diseases in war-affected communities are PTSD and depression, with prevalence rates much higher than in places without a recent history of open combat [3]. PTSD following traumatic events such as war and natural disasters happens globally, especially in low- and middle-income countries where mental health services are often least available [15]. PTSD increases the risk of developing physical health problems through sleep disturbances, interpersonal problems, parenting difficulties, and is most strongly associated with suicidal behavior, comorbid depression, and substance use [13]. It is also associated with decreased well-being and unemployment when it persists unremittingly for years in a subset of trauma-exposed survivors [16].

Even though effective psychological interventions for mass conflict survivors do exist, most low and middle income countries (LMICs) lack the resources to provide psychological treatments for all affected war survivors [4]. Therefore, more research is needed to inform the current and future mental health policies for LMICs to give their limited mental health services to the mental health needs of civilian war survivors in war-affected areas [17].

Many factors contribute to the development of post-traumatic stress disorder after exposure to traumatic events. For example, older age, female sex, potentially traumatic experiences during and after the war, unemployment, and a lower educational level are all linked to higher rates of PTSD [3].

According to a study on mental disorders following war applying multivariable analyses, older age, unemployment, female sex, more potentially traumatic experiences, low social class, substance use disorders, the nature of the trauma, witnessing someone being killed or seriously injured, and the demographic grouping of the survivor, including low socioeconomic status, being divorced, and being widowed, significantly increased the risk of experiencing traumatic events and were associated with PTSD [3, 12, 18].

Despite the fact that PTSD is common in post-disaster situations, limited research has been conducted on the war-affected area of Dessie Town. As a result of this research, mental health professionals could be able to provide interventions to improve their condition, treat people who are afflicted, and deal with life pressures. Finally, the findings of the present study could assist in contributing to the existing body of knowledge that requires further attention and suggest areas where further investigations are required. Therefore, this study aimed to determine the prevalence of PTSD and its associated factors among adult population residents following the war in Dessie Town.

## Methods

### Study area and study period

The study was conducted in Dessie town, South Wollo zone, Northeast Ethiopia, from 21 to 2022 to 21 July 2022.According to the Ethiopian official media announcement since July 2021, war attacks and abuse on towns and villages have occurred in northern Amhara, including Dessie. Hundreds of civilians were killed by shelling or massacred; over 550,000 people were displaced by mid-September 2021 as a result of the war [19].

Dessie Town is located 401 km far from Addis Ababa and 490 km from Bahir Dar. The town is organized in five sub-cities with 18 urban and eight rural kebeles (the smallest administration unit in Ethiopia). Dessie Town has 285,530 residents of those about 244,724 of residents in urban, 40,806 are rural residents according to Dessie city administration report in 2022.

### Study design

Community-based cross-sectional study.

### Population

All adult residents in Dessie Town were considered the source population, and adult residents in Dessie Town in selected households during the study period were the study population.

### Eligibility criteria

Residents over the age of 18 and those who had lived in Dessie Town for more than 6 months were included in the study, while those who were unable to provide proper information (unable to communicate) were excluded.

### Sample size determination

In this study, the sample size was determined by using a single population proportion formula based on the estimated prevalence rate of PTSD conducted in IDPs in South Ethiopia, which was 58.4% [20]. With a 95% confidence level, a 5% margin of error applying the formula, and a 10% non-response rate, the sample size was 410. Therefore, applying a design effect of 1.5, the final sample size was 615.

### Sampling procedure

**Fig. 1:**
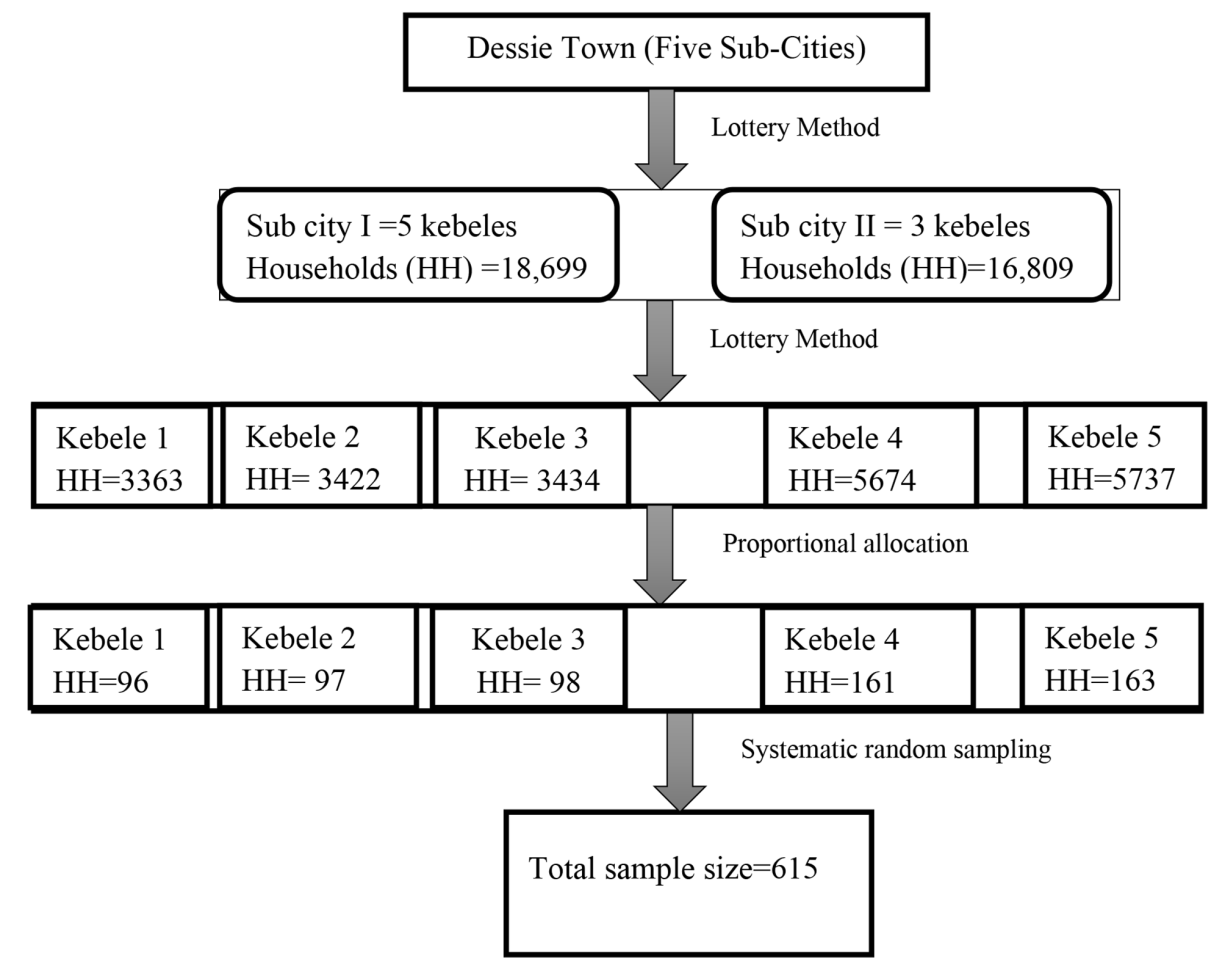
Schematic presentation of sampling procedure on the prevalence of post-traumatic stress disorder and associated factors among residents in the war-affected area, Dessie Town, Northeast Ethiopia, 2022

A multi-stage systematic random sampling method was used. Two sub-cities were randomly selected out of five sub-cities using the lottery method. Five kebeles were selected through the lottery method from a selected sub-city, and the sample size was distributed to the selected kebeles proportionally to the household size. Households in the selected kebele were selected by using systematic random sampling technique after identifying an initial starting household by lottery method (Fig. 1).

Eligible participants in the selected household were further selected and interviewed. In cases where there was more than one eligible participant in the household, the lottery method was used to include only one. When an eligible participant was not found at a particular time, the interviewer returned to the household three times at different time intervals, and if the interviewer was unable to locate the participant, the household was marked as a non-response. Then the next household was used.

### Operational definition

Depression: individuals scoring at the cut point of 10 or more out of 9 items on the patient health questionnaire [21].

Post-Traumatic Stress Disorder was measured by using 17 items of PCL- C with cut-off points of ≥ 50, which means adults who score < 50 have no PTSD and ≥ 50 have PTSD [22, 23].

Stressful life events: were measured using the presence of one or more of the life-threatening experiences questionnaires in the last six months [24].

Social support: - was measured using the Oslo 3-item social support scale (OSS-3), which has scores ranging from 3 to14. Poor social support is a score of “3–8” on OSS-3, Intermediate social support is a score of “9–11” OSS-3; strong social support is a score of “12–14” on OSS-3 [25].

Substance abuse is defined as the abuse of alcohol, khat, cigarettes, and illicit substances by respondents and fulfils the criterion (CAGE ≥ 2) [26, 27].

Experienced ill health without medical care: those participants who were experienced illness during the war but did not receive medical treatment at a time [28, 29].

### Data collection tools and procedures

Five BSc data collectors and two MSc supervisors were selected from the psychiatry profession, and training was provided to them by the principal investigator about data collection methods and tools. A pretest was conducted at Kombolcha Town on 31 individuals outside the study area for the clarity of the questionnaires two weeks prior to actual data collection. Psychiatric professionals translated a questionnaire to Amharic, and then it was translated back to English by an independent person to check for consistency and understandability of the tool. Regular supervision by the supervisors and the principal investigator was made to ensure that all necessary data were properly collected. The collected data was cleaned and checked for completeness before being processed and entered on paper into a computer.

A structured interviewer-administered questionnaire was used, which has six sub-sections: The Post-Traumatic Stress Disorder Civilian Version (PCL-C) was used to assess PTSD, which is an easily administered self-report rating scale for assessing the 17 DSM-IV symptoms of PTSD. A total score is computed by adding the 17 items, so that possible scores range from 17 to 85 with a five-point Likert scale( 1 = not at all, 2 = a little bit, 3 = moderately, 4 = quite a bit, 5 = extremely) with a cut point of ≥ 50 [22, 23]. This instrument was adapted from a Minnesota study on Somali and Oromo Ethiopians [23]. Internal consistency was found in community adults with values above 0.75 [30]. It has been utilized in Ethiopia [11].

Depression was measured by the Patient Health Questionnaire-9 (PHQ-9), which indicates depression if individuals score at cut point of 10 or more out of 9 items. It has 4 items consisting of: not at all (0), several days [1], more than half the days [2],, nearly every day [3], over the last 2 weeks [21]. It has been validated in Ethiopia to screen for the diagnosis of major depressive disorder among adults. It showed good internal Cronbach’s alpha = 0.81 with sensitivity = 86% and specificity = 67% [21].

The social support of patients was assessed using the Oslo social support scale (OSS-3). The OSS-3 has a sum score scale ranging from 3 to 14. A score of “3–8” on the OSS-3 indicates poor social support, “9–11” indicates intermediate social support, and “12–14” indicates strong social support (OSS-3) [25].

The List of Threatening Experiences (LTE) questionnaire was used to assess stressful events with the yes/no answers of respondents. The LTE consisted of 12 individual stressful event items. It is a valid and reliable measure of stress in mental health [24], and it has been utilized in Ethiopia [11].

The substance use history was assessed by ‘yes’ or ‘no’ question with ever and current use of the substance. In addition, the Cut down, Annoyed, Guilty, and Eye-Opener Questions Adapted to Include Drugs (CAGE-AID) questionnaire was used to screen for problematic substance uses (alcohol, khat, tobacco, and cannabis). Item responses on the CAGE questions were scored 0 for “no” and 1 for “yes” answers. A total score of two or greater positive answers from the four questions about social drugs (khat, alcohol, tobacco, and cannabis) was considered problematic substance use [26, 27].

A socio-demographic questionnaire used to assess the patients’ background information, clinical and trauma factors was assessed by the yes/no answers of respondents and operationalized according to different literature.

### Data processing and analysis

The data was checked for completeness, then entered into Epi-data version 4.6, and exported to SPSS version 25. The association between dependent and independent variables was assessed, and its strength was presented using adjusted odds ratios and 95% confidence intervals. Data was presented using frequency tables, charts, and figures.

Bivariable and multivariable logistic regression analyses were carried out. Variables with a p-value ≤ 0.25 were taken to multivariable analysis, and a p-value less than 0.05 was judged to be associated with PTSD. Model fitness (Hosmer and Lemeshow Test) was checked.

## Results

### Sociodemographic characteristics of the respondents

A total of 600 participants participated, with a response rate of 97.6%. The mean age of the respondents was 39.87 years, with a SD ± 10.063 years, and more than one third of respondents 222(37.0%) were between the ages of 26–35 years. More than half of the respondents were male 365(60.8%), married 439(73.2%), Amhara in ethnicity 574(95.6%), having a diploma or higher 230(38.3%), waged employees 257(42.8%), Orthodox 409(68.2%) and having a good income 386(64.3%) (Table 1).

**Table 1:** Sociodemographic characteristics of study participants among residents in the war-affected area, Dessie Town, Northeast Ethiopia, 2022. (n = 600)

Characteristic	Category	Frequency	Percentage
Age	18–25	15	2.5
	26–35	222	37.0
	36–45	199	33.2
	≥ 46	164	27.3
Sex	Female	235	39.2
	Male	365	60.8
Marital status	Married	439	73.2
	Single	84	14.0
	Divorced/ Widowed	77	12.8
Ethnicity	Amhara	574	95.6
	Tigre	12	2.0
	Oromo	13	2.2
	Afar	1	0.2
Educational status	No formal Education	155	25.8
	Primary schooling	114	19.0
	Secondary schooling	101	16.8
	Diploma and above	230	38.3
Occupational status	Waged employee	257	42.8
	Housewife	105	17.5
	Unemployed	63	10.5
	Self-employed	175	29.2

### Clinical and psychosocial factors

A total of 44(7.3%) participants have a known family history of diagnosed mental illness, and 63(10.5%) of the respondents have a known chronic medical condition that has been diagnosed. Concerning their current depression status, 192(32% study participants) reported depressive symptoms. Nearly one-third 218(36.3%) of the participants had ever used alcohol in their lifetime, and 187(31.2%) have currently used alcohol. The prevalence of problematic substance use in this study was 119(19.8%), 120(20%) of the participants had experienced forced separation from their family during the disaster, 48(8%) of the respondents had experienced trauma, neglect, rape in their childhood period, and nearly half (42.7%) reported having gone through at least one stressful life event in the previous six months, and 39.8% had strong social support (Table 2).

**Table 2:** Clinical and psychosocial factor of the respondents of residents in the war-affected area, Dessie Town, Northeast Ethiopia, 2022. (n = 600)

Clinical factors	Category	Frequency	Percentage
Known family history of diagnosed mental illness	Yes	44	7.3
	No	556	92.7
Known diagnosed chronic medical condition	Yes	63	10.5
	No	537	89.5
Type of medical condition	Diabetes Mellitus	15	2.5
	Hypertension	16	2.7
	HIV-Aids	12	2.0
	Cancer	4	0.7
	Heart Disease	11	1.8
	Others	5	0.8
Depressive Symptoms	Yes	192	32.0
	No	408	68.0
Ever khat used in lifetime	Yes	90	15.0
	No	510	85.0
Current khat used in the last 3 months	Yes	72	12.0
	No	528	88.0
Ever used alcohol drinks in life	Yes	218	36.3
	No	382	63.7
Current used alcohol drinks in last 3 months	Yes	187	31.2
	No	413	68.8
Ever used tobacco products	Yes	49	8.2
	No	551	91.8
Current tobacco products used in the last 3 months	Yes	40	6.7
	No	560	93.3
Problematic substance use	Yes	119	19.8
	No	481	80.2
Sexually abused or raped	Yes	29	4.8
	No	571	95.2
Had ill health without medical care	Yes	61	10.2
	No	539	89.8
Experienced forced separation from family	Yes	120	20.0
	No	480	80.0
Experienced trauma/raped in childhood	Yes	48	8.0
	No	552	92.0
Stressful Life Event	Yes	256	42.7
	No	344	57.3
Social Support	Poor Social Support	209	34.8
	Intermediate Social Support	152	25.4
	Strong Social Support	239	39.8

Others; Kidney Disease, Gastric Disease

### Factors associated with posttraumatic stress disorder

The prevalence of PTSD in these participants was 34.5% (95% CI; 31, 38). In the multivariable analysis, being female (AOR: 1.82, CI; 1.18, 2.82), divorced or widowed (AOR: 2.12, CI; 1.23, 3.66), learned their primary school(AOR: 2.17, CI; 1.25, 3.78), depressive symptoms (AOR: 2.03, CI; 1.34, 3.08), experiencing ill health without medical care during the disaster (AOR: 2.97, CI; 1.43, 6.16), forced separation from their family (AOR: 1.90, CI; 1.16, 3.12), experienced stressful life event (AOR: 1.60, CI; 1.06, 2.42)were found to be significantly associated with PTSD (Table 3).

**Table 3:** Bivariable and multivariable independent factors of PTSD among residents in the war-affected area, Dessie Town, Northeast Ethiopia, 2022(n = 600)

Variable	Category	PTSD Yes No		COR(95%CI)	AOR(95%CI)
Sex	Female	96	139	1.58(1.12, 2.23)	1.82(1.18, 2.82)**
	Male	111	254	1	1
Marital status	Married	144	295	1	1
	Single	21	63	0.68(0.40, 1.16)	0.56(0.30, 1.05)
	Divorced/Widowed	42	35	2.12(1.51, 4.02)	2.12(1.23, 3.66)**
Educational status	No formal education	45	110	0.9(0.56, 1.40)	1.53(0.86, 2.72)
	Primary education	51	63	1.78(1.12, 2.82)	2.17(1.25, 3.78)**
	Secondary education	39	62	1.38(0.85, 2.25)	1.49(0.84, 2.63)
	Diploma & above	72	158	1	1
Occupational status	Waged employee	105	152	1.55(1.03, 2.32)	1.31(0.81, 2.14)
	Housewife	31	74	0.94(0.55, 1.59)	0.62(0.32, 1.20)
	Unemployed	17	46	0.83(0.44, 1.57)	0.95(0.46, 1.96)
	Self-employed	54	121	1	1
Family history mental illness	Yes	22	22	2.01(1.08, 3.72)	0.96(0.46, 2.02)
	No	185	371	1	1
Depressive symptoms	Yes	100	92	3.06(2.14, 4.38)	2.03(1.34, 3.08)**
	No	107	301	1	1
Problematic Substance use	Yes	52	67	1.63(1.08, 2.46)	0.98(0.60, 1.59)
	No	155	326	1	1
Sexually abused or raped	Yes	19	10	3.87(1.77, 8.49)	1.15(0.42, 3.15)
	No	188	383	1	1
Ill health without medical care	Yes	43	18	5.46(3.06, 9.76)	2.97(1.43, 6.16)**
	No	164	375	1	1
Forced separation from family	Yes	68	52	3.21(2.13, 4.84)	1.90(1.16, 3.12)*
	No	139	341	1	1
Stressful life event	Yes	117	139	2.38(1.68, 3.35)	1.60(1.06, 2.42)*
	No	90	254	1	1

1 = Reference group, *p < 0.05; **p < 0.01, COR = Crude Odds Ratio, AOR = Adjusted Odds Ratio

## Discussion

PTSD is a significant health problem that can have serious consequences for patients, their families, and the community at large. This study showed that the prevalence of PTSD was 34.5% (95% CI: 31–38%) in the war-affected area of Dessie Town. This result was in line with other findings that reported a 32.3% lifetime prevalence of PTSD following seven years of trauma exposure in the Serbian community [31],, 34.9% in communities living in conflictual areas of Diyarbakir, Turkey [32], 36% among post-war conflict affected populations in Southern Sudan [33], and 37.3% among Koshe landslide survivors in Ethiopia [11].

However, our finding was lower than previous studies, that reported in Liberia during civil war conflict(48.3%) [34], and 38.46% in Syrian Kurdish refugees in Iraq [35]. This disparity could be attributed to the relentless continuation of intense conflict and war that lasted for many years, increasing adversity and devastation in Liberia. Thus, different studies indicated that the rate of PTSD increased as the war lasted for many years [34].

Likewise, the prevalence of PTSD in the current study was lower than in other studies, including 48% and 46% among Sudanese population and Sudanese refugees in Uganda and southern Sudan [36], 59.8% among survivors of the Maikadra massacre, and 58.4% among IDP in Ethiopia [37, 38]. This variation could be due to repeated exposure to the traumatic event, and having a prior displacement history could make them easily vulnerable to other displacement-related suffering and trouble [38].

On the other hand, our result was higher than the other studies, including 26.4% among survivors of the Kosovo War [39], 11.8% in Uganda [40], 28% in the war-affected population of South Sudan [10], and 19.4% among Dessie town residents [41]. The current study was carried out less than eight months after the start of the war, whereas other studies delayed carrying out the study after the war. The possibility of spontaneous remission from PTSD is decreased with further exposure to traumatic events, which raises the prevalence [42].

In this study, the odds of having PTSD were 1.82 times higher in females than in males. One possible explanation is that sexually traumatic events, such as rape and sexual abuse, are more commonly associated with PTSD in women [18]. This is supported by previous studies of war in Balkans [3], among IDP in Ethiopia [38] and Koshe landslide survivors in Ethiopia [11].

Participants who were separated/widowed/divorced were 2.12 times more likely to develop PTSD as compared with married respondents. The possible reason could be that those who have young children frequently worry about raising their family alone, and most people, especially those who have young children, tend to worry about money when facing life without a partner, which causes stress. This is consistent with the previous studies amongst Koshe landslide survivors in Ethiopia, Sudanese nationals refugees, in the West Nile and Kashmir Valleys in India, and, respectively [11, 36, 43].

Participants who had primary education (a few years of education) were 2.17 times more likely to have PTSD than those who had diploma or above. This could be due to lower educational levels associated with lower resilience, including poorer coping skills, lower self-esteem, and lower insight, among other things, causing victims to have difficulty recovering from trauma [44] .This is consistent with the study in northern Uganda and south Sudan among the general population and refugees [36].

The odds of developing PTSD were 2.03 times higher among individuals who had depressive symptoms when compared to respondents without depressive symptoms. This could be because participants with depressive symptoms are more likely to suffer traumatic stressors than respondents without depressive symptoms [45]. Depression might affect the risk of experiencing trauma and the progression to PTSD. This was supported by studies on IDP in Nigeria [46], and Maikadra massacre in Ethiopia [37, 38].

The odds of having PTSD were 2.97 times higher among people who had experienced ill health without medical care during the conflict than those who had not. This could be a result of anxiety sensitivity to react to chronic pain by catastrophically misinterpreting its meaning, physiologic arousal, fear of reoccurring pain, fear of movement or re-injury, and disability, which hurts a number of body systems if it persists for a long time [47]. Studies among IDPs in Nigeria and Uganda provide evidence in support of this [28, 29].

Participants who had experienced forced separation from family were 1.9 times more likely to have PTSD than those who had not experienced this event. This could be because family separation affects how well people recover psychologically from trauma when there is constant, forced separation from important attachment figures. In addition, this sense of loss may reduce the ability of the attachment system to function correctly, which could change how the brain regulates itself in the presence of perceived threats and stress, making people less able to handle daily pressures [48].This is supported by a study of the post-war conflict-affected population in Southern Sudan [33].Participants who had experienced stressful life events were more likely to have PTSD than those who had not. Stressful life events, such as problems with work, relationships, or finances, can exacerbate PTSD [49].

In summary, these findings highlight the significant impact of war and conflict on mental health, particularly PTSD. The high prevalence of PTSD and its association with various sociodemographic, clinical, and psychosocial factors underscores the need for comprehensive mental health services and interventions in war-affected areas. Such services should be sensitive to the specific needs and experiences of different demographic groups, particularly women, those with lower educational attainment, and those with a history of trauma or mental illness.

### Limitations of the study

People without PTSD may be less motivated to recall earlier exposures than those who do, which could explain recall bias. There may be a bias in memory or information for stressful life events due to the overlap between PTSD symptoms and those resulting from stressful life events, regardless of time. Social desirability bias may also be a problem, as the participants may tend to provide socially acceptable responses to sensitive questions related to sexual abuse and substance use.

## Conclusion

Our findings show that there is a high prevalence of PTSD among residents of Dessie Town, a war-affected area. Factors that contribute to PTSD include being female, divorced/widowed/separated, having primary schooling, depression, lack of medical care for ill health, forced separation from family, and experiencing stressful life events. To reduce PTSD symptoms, post-disaster interventions are needed to help residents cope with the stress of war trauma. The concerned organization may then put these approaches into action to help minimise the occurrence of PTSD among war survivors via early detection, prevention, and intervention. In addition, we advised the state administration to implement a planned psychosocial intervention among the people to enhance living conditions and address the effects of traumatic stress. We also promoted the establishment of a weekly mental health clinic in the neighbourhood to assist locals experiencing mental distress.

## Data Availability

The data sets of the current study are available from the principal author [Zelalem Birhan].

## Abbreviations

AOR: Adjusted Odds Ratio
BSc: Bachelor of Science
CI: Confidence Interval
COR: Crude Odds Ratio
HTQ: Harvard Trauma Questionnaire
LMIC: Low Middle Income Countries
LTE: List of Life-Threatening Experiences
M.I.N.I.: Mini International Neuropsychiatric Interview
MSc: Master of Science
OSSS: Oslo Social Support Scale
PCL-C: Post-Traumatic Stress Disorder Civilian Version
PHQ-9: Patient Health Questionnaire
PTSD: Post-Traumatic Stress Disorder
SD: Standard Deviation
